# Association between sexual dysfunction and vitamin D in Swedish primary health care patients born in the Middle East and Sweden

**DOI:** 10.1038/s41598-023-50494-6

**Published:** 2024-01-05

**Authors:** Marina Taloyan, Kristin Hjörleifdottir Steiner, Claes-Göran Östenson, Helena Salminen

**Affiliations:** 1 Division of Family Medicine and Primary Care, Department of Neurobiology, Department of Neurobiology, Care Sciences and Society (NVS), Karolinska Institutet, Alfred Nobels allé 23, 14183 Stockholm, Sweden; 2 Academic Primary Health Care Centre, Region Stockholm, Box 45436, SE-104 31 Stockholm, Sweden; 3 Endocrine and Diabetes Unit, Department of Molecular Medicine and Surgery, Karolinska Institutet, Stockholm, Sweden

**Keywords:** Endocrinology, Health care, Medical research

## Abstract

The present study investigated primary care patients and compared self-reported sexual health in Swedes and Middle Easterners; analysed differences within and between the groups and analysed differences in 25-hydroxyvitamin D [25(OH)D] levels between the groups. 522 patients responded to a health questionnaire that included items on sexual health: 225 Middle Easterners from Iran, Iraq, and Turkey and 297 Swedes. Logistic regression was used to calculate the odds ratio (OR). Middle Easterners reported less sexual dysfunction than Swedes, and 75.8% of them and 18.9% of Swedes presented a 25(OH)D of < 50 nmol/L. The crude OR for reporting sexual dysfunction was 70% higher in Swedes compared to Middle Easterners (OR 1.70, 95% CI 1.15–2.50). This OR remained significant after adjustment for age, gender, waist circumference, and reported sexual desire. However, the significance disappeared after additional adjustment for 25(OH)D. In both groups, more females than males reported insufficient sexual desire. More female Middle Easterners reported sex life dissatisfaction. More female Swedes reported sexual dysfunction. Vitamin D could explain an association between gender and sex life dissatisfaction in Middle Easterners, and age could explain an association between gender and sexual dysfunction in Swedes. Age, waist circumference, and 25(OH)D levels were significant covariates in the logistic regression models. Results from the present study suggest that 25(OH)D variation partly explains differences in sexual dysfunction between the groups and between genders within the groups. Vitamin D therapy should be investigated to determine if these results are clinically useful.

## Introduction

Sexuality is an essential part of being human and is important for quality of life^1^. Impaired sexual function is a clinical condition^1–3^. Loss of sexual desire is a primary disorder^1–3^ and is not caused by other problems such as erectile dysfunction, dyspareunia, or other diseases. Hormonal changes in females and males due to aging^2^ and psychosocial factors^3^ may play a role in the loss of sexual desire. Sexual disorders occur as a consequence of obesity in males of all ages^4^.

The prevalence of specific, sexual dysfunction manifestations varies considerably^5–7^ for females and males. Epidemiological studies of male sexual dysfunction consistently indicated that dysfunction increases with age. Sexual dysfunction increases by 10% in males aged 40–49 and 100% in males aged 70–89^4,5^. Additionally, male sexual desire decreases in advanced age^8,9^. Female sexual dysfunction also increases in advanced age. And female sexual dysfunction varied up to 50%^6–8,10–12^.

Studies of sexual dysfunction in the UK reported a higher prevalence of the disorder in males of South Asian descent than in males of European descent in the UK^13^. Similarly, another study in London concluded that those, who sought care for dysfunction, came mainly from Asia, the Middle East, or North Africa^14^. A Swedish study of patients with type-2 diabetes found no differences in sexual dysfunction prevalence. That said, the study reported a higher prevalence of decreased sexual desire (self-reported) in Assyrians than in Swedes^15^.

Some evidence suggests that vitamin D can affect sexual health. A previous clinical trial that included males aged 35–64 years with normal testosterone levels at baseline found that vitamin D treatment in males with vitamin D deficiency could increase testosterone levels and improve erectile function^16^. According to an overview, low levels of 25-hydroxyvitamin D [25(OH)D] might lead to a decrease in erectile function via several mechanisms, such as regulation of endothelial function, intervention in inflammatory conditions, interfering with hypertension, type 2 diabetes, kidney disease, and abnormal cholesterol levels^17^. It is shown that sexual dysfunction in patients with dialysis treatment is associated with vitamin D deficiency^18^. A recent meta-analysis concluded that vitamin D treatment can prevent diabetes disease with erectile function^19^. A pilot study of young females found a correlation between vitamin D deficiency and lower self-rated sexual health as evaluated by the *Female Sexual Function Index*—particularly in the areas of sexual desire, orgasm dysfunction, and sexual satisfaction^20^.

Vitamin D deficiency prevalence varies between immigrants to Europe and native-born Europeans. A German study reported that 74% of Turkish immigrants (male and female) had a vitamin D deficiency—compared to 33% of native Germans (defined as 25(OH)D of < 30 nmol/L). Deficiency prevalence was somewhat higher in Turkish females, who wore ethnic skin-covering clothing, than females who wore Western clothing (86% vs. 69%)^21^.

While 25(OH)D variation between native Europeans and Middle Eastern immigrants was found, no data or analyses indicate whether this variation is associated with differences in sexual health. If this association exists, it might imply that immigrants seeking care for sexual dysfunction should be tested, and when appropriate, treated for vitamin D deficiency. So, this study aimed to (*i*) determine if a difference in sexual health exists between Swedes and Middle Easterners (self-reporting, primary care patients) and (*ii*) determine if any existing differences are associated with 25(OH)D variation.

## Materials and methods

### Study population

Data used in the present study came from (*i*) “Screening and treatment of prediabetes in primary health care: a pilot study” and (*ii*) “Health problems and vitamin D status in primary health care patients from different ethnic groups”. The former (4-D diabetes study; 2013–2015) was done at the Flemingsberg and Jakobsberg Primary Health Care Centres in Stockholm (PHCC). The latter (vitamin D study; 2015–2016) was done at the Flemingsberg PHCC. In total 830 participants between the ages of 18–74 years were interviewed and investigated in the 4D study and 99 participants in Vitamin D study. The specific healthcare centres were chosen due to their high proportion of non-Swedish born in the population, with Flemingsberg’s PHCC having approximately 60% of their inhabitants born outside of Europe. Individuals born in Middle East, Africa and Asia were eligible for both studies (4D and Vitamin D). Patients with a previous diagnosis of diabetes mellitus and/or severe psychiatric illness were excluded from the 4D Diabetes project.

Inclusion criteria for both studies were (*i*) being born in Sweden (persons born in Sweden whose parents were also born in Sweden and are of northern European descent) and (*ii*) being born in the Middle East, Africa, or Asia (an immigrant in Sweden). The 4-D study excluded patients with a diabetes diagnosis.

All patients, who consecutively sought primary care during the relevant periods and met the inclusion criteria, received a verbal invitation to participate in the studies. In total, 929 patients were recruited to both studies at the Flemingsberg PHCC (n = 394 in the 4-D study and n = 99 in the vitamin D study) and the Jakobsberg PHCC (n = 436 in 4-D study). Patients (n = 23), who participated in both studies at the Flemingsberg PHCC, were only counted once. So, in total, 903 patients participated in both studies. Firstly, the patients were asked by either the reception or assistant nurses in the waiting room to participate short interviews for identification of eligible participants in the study. Patients got written and oral information about the project and were informed that participation is voluntary and could be terminated at will. Written consent was attained prior to the start of the interview. Patient interviews were conducted in a separate room and were recorded if the patients consented to it. Patients responded to a questionnaire about their health by assistant nurses and translator if needed. At the second visits patients provided blood samples for testing of blood glucose levels (HbA1C) which were sent to Karolinska University Hospital. The participants were asked additional questions regarding their lifestyle, nutrition, socioeconomic factors, general health assessments^22^, self-reported sexual health^15^ and Finnish Diabetes risk assessment scale (FINDRISC)^23^ diabetes risk assessment validated and used in peer-reviewed research studies. Questions about sexual health were used for first time in study investigated sexual health in patients with type 2 diabetes resettled in a Swedish town Södertälje^15^.

The present study included patients of northern European descent who were born in Sweden and whose parents also were born in Sweden (n = 297) and patients of Middle Eastern descent born in Turkey, Iran, or Iraq (n = 225), namely, 522 patients in total.

### Independent variables

First independent variable was *Origin* categorized as Swedes and Middle Easterners; see above.

### Dependent variables

Overall *self-reported sexual health* and:

- *Sexual function* based on “Can you complete the act of intercourse?”—Item defined as the ability to have intercourse (yes/no);
- *Sexual desire* based on “Do you have any sexual desire?”—Item dichotomized as either present or absent (yes/no); and
- *Sex life dissatisfaction*: “How satisfied are you with your sex life?”—Item responses on a five-point scale: very satisfied, somewhat satisfied, neutral, somewhat dissatisfied, very dissatisfied.

### Covariates

The present study included these covariates: age, sex, birthplace, BMI, waist circumference and Vitamin D *levels* (25(OH)D in nmol/L). *Age* in years was a continuous variable. *BMI* was continuous and calculated as weight divided by height squared (kg/m^2^) and grouped as: normal (BMI < 25); overweight (BMI ≥ 25 and < 30); and obese (BMI ≥ 30). *Waist circumference* in cm was a continuous variable. Vitamin D *levels* (25(OH)D in nmol/L) was categorized as: normal (25(OH)D ≥ 50 nmol/L); inadequate (25(OH)D ≥ 25 and < 50 nmol/L); and deficient (25(OH)D < 25 nmol/L)^15^.

The present study selected 25(OH)D levels to analyze because (*i*) it is the metabolite of vitamin D that is used for determining a person’s vitamin D status; (*ii*) it has a long half-life, and (*iii*) its production by the liver is not significantly regulated—versus production of 1,25(OH_2_)D by the kidneys^24,25^. In addition, circulating 1,25(OH_2_)D has only one primary role—regulation of serum calcium concentrations. Most 1,25(OH_2_)D is generated by extrarenal production as needed by various organs^26^.

### Statistical methods

Differences in normally distributed variables between Swedes and Middle Easterners were determined with a Student’s t-test. Categorical variables were gender, sexual function, sexual desire, and sex life satisfaction. Statistical significance—of differences in these variables between Swedes and Middle Easterners—was determined with a chi-square test. Logistic regression revealed the odds ratios between Swedes and Middle Easterners and separately, between females and males in each group. Stepwise adjustment for each explanatory variable was presented in models; each model was added stepwise to the previous model. Dependent variables that were not significantly different in studied groups (Swedes and Middle Easterners) were including in the logistic regression models as covariates. A p-value of less than 0.05 was considered statistically significant. STATA 14 software (StataCorp LP, Texas) was used for all statistical analyses.

### Ethics-approval and consent to participate

An ethical permit (review number 2013/2303–31/3). was granted by the Regional Ethical Review Board in Stockholm. Written informed consent was obtained from participants. To maintain the privacy of the participants was the data coded. All study information was translated from Swedish into three languages: Turkish, Farsi, and Arabic. Subsequently, abnormal blood test results and/or diabetes were scheduled for further investigation and regular follow-ups at the health care centre. Relevant guidelines and regulations (e.g. Declaration of Helsinki) were followed in all methods.

## Results

### Demographic characteristics

The present study had 522 patients 297 Swedes (56.9%) and 225 Middle Easterners (43.1%); see Table 1. Females were in the majority among Swedes (58.3%) and Middle Easterners (59.6%). Middle Easterners were significantly younger than Swedes with (mean ages 45.3 and 55.0, respectively). Statistically significant differences between the two groups were found in median 25(OH)D levels of 67.4 nmol/L in the Swedes and 37.0 nmol/L in the Middle Easterners. Reporting of sexual dysfunction was significantly higher among Swedes.

**Table 1:** Characteristics of the participants in the study based on the region of birth.

	All patients	Swedes	Middle Easterners	P-value
Total (%)	522 (100%)	297 (56.9%)	225 (43.1%)	
Age (years)				
Mean (SD)	50.8 (0.63)	55.0 (0.87)	45.3 (0.78)	** < 0.0001**^**a**^
Waist circumference				
Mean (SD)	98.0 (0.64)	99.6 (0.86)	95.3 (0.93)	**0.004**^**a**^
25(OH)D (nmol/L)				
Mean (SD)	53.7 (1.5)	67.4 (1.9)	37.0 (1.6)	** < 0.0001**^**a**^
25(OH)D (nmol/L)				
< 25	69 (13.2%)	8 (2.7%)	61 (27.1%)	** < 0.0001**^**a**^
25–49.9	135 (25.9%)	48 (16.2%)	87 (38.7%)	
≥ 50	318 (60.9%)	241 (81.1%)	77 (34.2%)	
Gender				
Male n (%)	215 (41.2%)	124 (41.8%)	91 (40.4%)	0.76^b^
Female n (%)	307 (58.8%)	173 (58.3%)	134 (59.6%)	
Sexual function				
Yes (n,%)	145 (28.8%)	69 (24.1%)	76 (35.0%)	0.008^b^
No (n,%)	358 (71.2%)	217 (75.9%)	141 (65.5%)	
Sexual desire				
Yes (n,%)	125 (38.7%)	109 (38.0%)	86 (39.6%)	0.70^b^
No (n,%)	209 (61.3%)	178 (62.0%)	131 (60.4%)	
Sex life satisfaction				
Yes (n,%)	240 (52.2%)	133 (53.2%)	107 (51.0%)	0.63^b^
No (n,%)	220 (47.8%)	117 (46.8%)	103 (49.0%)	

^a^Student’s t-test, ^b^Chi-square.

No statistically significant differences occurred regarding sexual satisfaction and sexual desire between the groups. The most striking difference was in vitamin D level: 227 Swedes (81.6%) and 66 Middle Easterners (35.5%) had a normal 25(OH)D level of ≥ 50 nmol/L (p < 0.0001).

Table 2 presents gender differences regarding sexual health. Significant gender differences occurred regarding (*i*) insufficient sexual desire and sexual dysfunction in Swedes and (*ii*) sex life dissatisfaction in Middle Easterners. Compared to male Swedes, female Swedes more frequently reported insufficient sexual desire (52.3% versus 47.7%) and sexual dysfunction (60.4% versus 39.6%), which is significant. Female Middle Easterners reported significantly more sex life dissatisfaction than male Middle Easterners (67.0% versus 33.0%, respectively).

**Table 2:** Prevalence of sexual health by gender among Swedes and Middle Easterners.

Covariates	Swedes			Middle Easterners		
	Females	Males	Chi^2^ test	Females	Males	Chi^2^ test
Total population, (%)	134 (59.6)	91 (40.4)		173 (58.3)	124 (41.7)	
Self-reported sexual desire, n (%)	93 (52.3)	85 (47.7)	**0.03**	70 (53.4)	61 (46.6)	0.06
Self-reported sexual function, n (%)	131 (60.4)	86 (39.6)	**0.04**	83 (58.9)	58 (41.1)	0.89
Self-reported sex life satisfaction, n (%)	63 (53.9)	54 (46.1)	0.60	69 (67.0)	34 (33.0)	**0.01**

### Logistic regression

Table 3 displays the difference in sexual dysfunction in Swedes (with Middle Easterners as reference). The crude OR of reporting sexual dysfunction in Swedes patients was 1.70 (95% CI 1.15–2.50). The adjustment for age and sex increased the odds ratios to 2.14. Stepwise inclusion in the logistic regression models of waist circumference and sexual desire slightly changes the odds ratios (2.17 and 2.11). In the final model with the inclusion of the vitamin D level covariate, the odds ratios changed to non-significant, OR = 1.50 (95% CI 0.92–3.23). Age, waist circumference, and 25(OH)D levels were significant covariates. Patients who had adequate 25(OH)D levels had 19 times higher odds of reporting sexual dysfunction than those with 25(OH)D levels under 25 nmol/L (OR = 19.35, 95% CI 8.50–43.90).

**Table 3:** Associations between sexual dysfunction and covariates variables in Swedes with Middle Easterners as a reference.

Variable	Crude	Model 1^a^	Model 2^b^	Model 3^c^	Model 4^d^
		OR 95%	OR 95%	OR 95%	OR 95%
Group					
Middle Easterners	Reference	Reference	Reference	Reference	Reference
Swedes	**1.70** (1.15–2.50)	**2.14** (1.00–2.40)	**2.17** (1.15–2.50)	**2.11** (1.15–2.50)	1.50 (0.92–3.23)
Age		**1.01** (1.04–1.07)	**1.01** (1.04–1.07)	**1.05** (1.04–1.07)	1.04 (0.98–1.01)
Gender					
Female		Reference	Reference	Reference	Reference
Male		1.01 (1.04–1.07)	1.01 (1.03–1.07)	0.81 (0.53–1.23)	0.91 (0.55–1.52)
Waist circumference		**1.01** (1.00–1.03)	**1.01** (1.00–1.03)	**1.01** (1.00–1.02)	**1.03** (1.00–1.04)
Sexual desire					
Yes				Reference	Reference
No				1.06 (0.67–1.68)	1.40 (0.81–2.40)
25(OH)D					
< 25 nmol/L					Reference
25–49.9 nmol/L					**3.30** (1.41–7.80)
≥ 50 nmol/L					**19.35** (8.50–43.90)

Significant odds ratios are in bold.

^a^Crude + age, sex; ^b^Model 1 + waist circumference; ^c^Model 2 + sexual desire; ^d^Model 3 + 25(OH)D.

Because gender differences occurred regarding insufficient sexual desire, more statistical analyses were done to explore possible covariant influences. Table 4 displays the crude odds ratio for female Swedes (with male Swedes as reference). Female Swedes had a crude odds ratio of 1.71 for insufficient sexual desire; it rose to 1.87 after adjustment for age. So, females had the probability of reporting insufficient sexual desire 87% more times than male Swedes.

**Table 4:** Associations between insufficient sexual desire and covariates in female Swedes with male Swedes as reference.

Variable	Model 1^a^	Model 2^b^	Model 3^c^	Model 4^d^	Model 5^e^
	OR (95% CI)	OR (95% CI)	OR (95% CI)	OR (95% CI)	OR (95% CI)
Group					
Middle Easterners	Reference	Reference	Reference	Reference	Reference
Swedes	**1.87** (1.13–3.10)	**1.83** (1.08–3.11)	**2.40** (1.32–4.30)	**2.18** (1.20–4.02)	**2.17** (1.05–3.95)
Age	1.02 (1.00–1.03)	1.01 (1.00–1.03)	1.01 (1.00–1.03)	1.02 (1.00–1.04)	1.01 (1.00–1.02)
Waist circumference		**1.05** (1.03–1.07)	**1.05** (1.03–1.07)	**1.05** (1.03–1.07)	**1.05** (1.02–1.07)
Sexual function					
Yes			Reference	Reference	Reference
No			**2.43** (1.25–4.71)	1.78 (0.88–3.62)	1.62 (0.74–3.60)
Sexual dissatisfaction					
No				Reference	Reference
Yes				1.05 (0.60–1.85)	0.80 (0.42–1.50)
25(OH)D					1.00
					(0.98–1.00)

Crude model (OR = 1.71, 95% CI (1.04–2.80).

Significant odds ratios are in bold.

^a^Model 1 + age; ^b^Model 2 + waist circumference; ^c^Model 3 + sexual function; ^d^Model 4 + sexual dissatisfaction; ^e^Model 5 + 25(OH)D.

Adjustment for differences in age and waist circumference and sexual function increased the odds ratios from 1.87 to 2.40. In the final model, with the inclusion of 25(OH)D levels, the odds ratios were almost the same (2.17) and still significant.

Results of logistic regression analyses of differences in reporting sexual dysfunction between female Swedes with male Swedes showed that in the crude model, male Swedes had 57% higher odds of having sexual desire—compared to female Swedes. In the age-adjusted model, the odds ratios decreased to nonsignificant levels (not shown in the table).

Table 5 displays the results of the logistic regression analyses for reporting sex life dissatisfaction among female Middle Easterners—compared to male Middle Easterners. The odds ratio for females was more than twice that for males in the crude model (OR = 2.07). When differences in 25(OH)D levels were accounted for in the final model, the odds ratio for reporting sex life dissatisfaction in females decreased and was no longer significant. So, differences in vitamin D levels could explain gender differences in reporting sex life dissatisfaction. The sexual desire covariate was the only significant one: regardless of gender, those who reported insufficient sexual desire had 5 times higher odds of reporting sex life dissatisfaction than those who did *not* report insufficient sexual desire.

**Table 5:** Associations between sex life dissatisfaction and covariates in female Middle Easterners with male Middle Easterners as reference.

Variable	Crude	Model 1:	Model 2:	Model 3:	Model 4:
	OR (95% CI)	OR (95% CI)	OR (95% CI)	OR (95% CI)	OR (95% CI)
Group					
Males	Reference	Reference	Reference	Reference	Reference
Females	**2.07** (1.18–3.61)	**2.09** (1.20–3.70)	**2.09** (1.20–3.70)	**2.10** (1.20–3.80)	1.77 (0.90–3.00)
Age		1.00 (0.98–1.03)	1.00 (0.98–1.03)	1.01 (0.99–1.04)	1.02 (0.99–1.06)
Sexual function					
Yes			Reference	Reference	Reference
No			0.90 (0.50–1.62)	0.50 (0.24–1.06)	0.57 (0.22–1.46)
Sexual desire					
Yes				Reference	Reference
No				**2.81** (1.32–6.00)	**5.30** (2.06–13.60)
25(OH)D status					
≥ 50 nmol/L					Reference
25– 49.9 nmol/L					1.10 (0.51–2.34)
< 25 nmol/L					0.85 (0.35–2.10)

Significant odds ratios are in bold.

^a^ Crude + age, sex; ^b^Model 1 + sexual function; ^c^Model 2 + sexual desire; ^d^Model 3 + 25(OH)D.

## Discussion

The present study investigated primary care patients (Swedes and Middle Easterners). It analysed differences within and between the groups regarding (*i*) sexual health factors and (*ii*) differences in 25(OH)D levels.

One-third of all patients reported sexual dysfunction. Compared to Swedes, Middle Easterners had significantly *lower* sexual dysfunction prevalence and odds of having a sexual dysfunction. After adjustment for all covariates and vitamin D, differences between the groups changed to nonsignificant. This suggests that vitamin D status might help explain the observed difference in probability regarding sexual dysfunction. No significant differences existed between Swedes and Middle Easterners regarding self-reported sexual desire or sex life satisfaction.

Results of the present study were unexpected regarding reporting worsen sexual life in Swedish-born than in Middle Eastern-born patients and not aligned with the results of other studies. Malavige et al. reported no significant difference in erectile dysfunction (ED) prevalence between south Asians and Europeans and premature ejaculation was significantly more common in south Asian males. That said, the present study and this study may not be comparable due to differences in population and methods^13^. Another study of males from the Middle East and South Asia reported that most patients who sought care at a clinic in London were of north African, Middle Eastern, or south Asian descent^14^. Both studies involved males only, which is not similar to the present study included males and females.

A Swedish study with a sample comparable to the present study found a higher prevalence of decreased sexual desire in Assyrians than in Swedes—and no differences in sexual function. Although the study was methodologically similar to the present study, it only included patients with type-2 diabetes^15^.

As already mentioned in the Introduction, sexual dysfunction and lower self-rated sexual health has been noted in different people with vitamin D deficiency^16,19,20^.

Other studies reported a higher prevalence of vitamin D deficiency in Middle Easterners living in Europe than in native Europeans^21,27^, and the present study confirmed this finding. This isn’t surprising because the evidence in this area is strong. Skin colour is one but not the only potential cause of this difference. Moreno-Reyes et al.^27^ reported that Moroccans and Turks residing in Brussels had higher vitamin D levels than residents from the Congo—even though the Congolese had darker skin colour. These researchers suggested that it might be due to factors such as socio-economic status and UVB light exposure rather than differences in diet because few food items contain significant amounts of vitamin D. The present study only included Middle Easterners.

Another study with 1231 participants reported higher vitamin D deficiency prevalence in Turkish immigrants than in ethnic Germans—and even higher prevalence in Turkish females who covered their bodies^21^. This finding suggests that limiting the amount of sunlight that reaches the skin (with clothing) can affect vitamin D levels^21^. And while the present study did not investigate clothing, the populations are comparable, and the present study yielded a similar result.

The present study also found significant gender differences regarding insufficient sexual desire and sexual function among Swedes. Age explained differences between genders (Swedish) in those who reported sexual dysfunction. Covariates could not explain gender differences regarding insufficient sexual desire among Swedes nor could they explain the probability of reporting insufficient sexual desire.

The discrepancies call for further investigation. Explanations could be found in a deeper investigation of hormonal disturbances or mental health. But among Middle Easterners, those with lower 25(OH)D levels had a significantly lower risk of reporting insufficient sexual desire than those with normal 25(OH)D levels. This was unexpected but might be explained by perceptions about sex life and mental health rather than the physiological pathology. Further, deeper investigations of sexual desire among Middle Easterners are also warranted.

A growing number of studies reported age-related increases in sexual dysfunction in males—largely attributable to performance disturbances due to atherosclerosis^8,9,28^. Consequently, in the present study, age was expected to be a significant covariate.

Multimorbidity and polypharmacy in older persons might explain this—as might be psychological issues. In addition, the present study’s population had risks for developing type-2 diabetes—a disease associated with fatigue, depression, and other health conditions that affect sex life.

### Strengths and limitations

The material used in the present study consists of data that was the first of its kind to be gathered in Sweden. The aforementioned *4-D diabetes* and 25(OH)D studies were the first to compare sexual health factors among Swedes and Middle Easterners. Covering so many factors can be a strength—if a general overview of health is the aim. But it may also be a weakness because the questionnaire only briefly dealt with sexual health.

Results from the present study imply that a greater prevalence of low 25(OH)D levels among Middle Easterners could be associated with a higher risk for sex life dissatisfaction. A Deleskog et al. study suggested that 25(OH)D levels can trigger sex-life dissatisfaction—a common complaint among patients with type-2 diabetes. Not unlike patients in the Deleskog study, patients in the present study had a higher risk of developing type-2 diabetes. And this suggests that they should be tested for 25(OH)D levels—as a proxy for type-2 diabetes within 10 years—and be subscribed to vitamin D for type-2 diabetes prevention, which the Deleskog study recommended^29^. The Deleskog study included a non-immigrant population resettled in Stockholm and the results were that those with prediabetes had lower 25(OH)D levels and higher diabetes risk as measured after 10 years. However, because this was a cross-sectional study, it was not possible to determine causality, i.e., whether the difference in 25(OH)D levels caused the difference in reporting declined sexual health, in general, and sex life dissatisfaction. Instead, causes not included in the statistical analysis, such as differences in diet, health conditions, amount of exercise, or measurement of 25(OH)D levels during different seasons (winter or summer) may explain the findings.

The previously published studies of patients on hemodialysis concluded that treatment with vitamin D did not improve sexual function in patients with vitamin D deficiency^18,30^. But this could be because of the patient’s other medical condition. One study investigated the treatment of 102 healthy middle-aged males with vitamin D deficiency and ED with a high dosage of ergocalciferol, a form of vitamin D, over at least 12 months. The men’s ED improved, their testosterone increased, and their HbA1c decreased^17^. The researchers also observed a small decrease in the men’s BMI^16^. Knowledge of this area is insufficient and further studies on the effects of treatment with different forms of vitamin D are highly needed.

Another limitation of the present study was the type of questions asked. Because of the nature of the two studies from which the data for this study were collected (they were general overviews of the participants’ health), the questionnaire only briefly touched on sexual health with three issues: ability, desire, and satisfaction. For example, impaired sexual function consists of one single yes/no question without detailed categorization into its specific aetiologies (e.g., ED, dyspareunia). A fuller investigation of the topic would require additional questions.

The present study included patients from the countries of origin of large immigrant groups in Sweden. This makes the results of the study readily applicable to a large part of the Swedish immigrant population. But the study sample may not be representative of immigrants who came to Sweden very recently—many of whom come from Syria and Afghanistan. Although it may be plausible to assume that the results of the present study may apply to immigrants from Syria (because of the geographic proximity of Syria to countries included in the present study), it is less likely that they apply to immigrants from Afghanistan, due to geographic distance and perhaps cultural and religious differences.

The skewed sex distribution in the present study’s population (58.8% female) may make the results more applicable to females than males. It also makes it harder to compare the present study’s results to those of other studies, because many previous studies investigated ED and vitamin D. That said, the gender distribution of the Swedes and the immigrants did not vary significantly in the present study; consequently, the skewed distribution did not affect the comparison between these two groups—as seen when gender was add in the logistic regression. Moreover, other possible confounders such as education and socioeconomic status were not including in the analysis which is an additional limitation in this study.

Some factors may render the present study’s population as not representative of the entire population of Sweden because it is based on primary care patients. The present study lacks information regarding patients who *refused to participate* in the aforementioned *4-D diabetes* and 25(OH)D levels. Only patients seeking care at a PHCC and only those who accepted to participate were included, which means that people who were healthy or for some other reason did not seek primary health care were not included in the studies. The study population cannot be considered representative of the population in Sweden at their age ranges as immigrants are in general younger that native population^31^. However, compared to the general population, the present study’s population may contain a larger proportion of people with chronic health conditions that can affect sexual health. Recruitment occurred in two areas outside Stockholm with high immigrant populations, which might make the results more difficult to apply to the population of other areas in Sweden. Recruitment at multiple geographic locations—and not limited to patients—is warranted for future studies.

Although the results of the present study may not be applied clinically because the study did not establish any kind of causality, it provides ideas for future clinical studies that could investigate possible vitamin D treatment effects. The present study lacked data on whether participants in the *4-D diabetes* study were treated with vitamin D supplements. If found effective in such studies, vitamin D supplementation could contribute to treatment for sexual health problems. It might also have positive effects on other aspects of health because other symptoms are linked to vitamin D deficiency^32,33^.

## Conclusions

The present study achieved its objectives to investigate primary care patients (Swedes and Middle Easterners), comparing their self-reported sexual health status and analyse differences in 25(OH)D vitamin levels between the groups. Study found significant differences in sexual health within studied groups but not between the groups. However, as expected, 25(OH)D levels were significantly lower in patients born in Middle East. The study also revealed that 25(OH)D variation partly explains differences in sexual dysfunction between the groups and between genders within the groups. However, the study cannot establish causality. Investigations of vitamin D treatment should be done to determine whether these results are clinically useful. The present study’s findings might be explained by perceptions about sex life and mental health—rather than physiological pathology. More extensive investigations of sex life for female Swedes and Middle Easterners are warranted.

## Data Availability

Neither data nor materials are publicly available as sharing the data with other researchers was not included in written informed consent. The first author (MT) is the contact person in case of making a request.
